# Variable Fitness Response of Two Rotifer Species Exposed to Microplastics Particles: The Role of Food Quantity and Quality

**DOI:** 10.3390/toxics9110305

**Published:** 2021-11-13

**Authors:** Claudia Drago, Guntram Weithoff

**Affiliations:** 1 Department for Ecology and Ecosystem Modelling, University of Potsdam, 14469 Potsdam, Germany; weithoff@uni-potsdam.de; 2 Berlin-Brandenburg Institute of Advanced Biodiversity Research (BBIB), 14195 Berlin, Germany

**Keywords:** microplastics, population growth rate, polystyrene, polyamide, silica beads, fitness response, rotifers, *Brachionus fernandoi*, *Brachionus calyciflorus*, egg ratio

## Abstract

Plastic pollution is an increasing environmental problem, but a comprehensive understanding of its effect in the environment is still missing. The wide variety of size, shape, and polymer composition of plastics impedes an adequate risk assessment. We investigated the effect of differently sized polystyrene beads (1-, 3-, 6-µm; PS) and polyamide fragments (5–25 µm, PA) and non-plastics items such as silica beads (3-µm, SiO_2_) on the population growth, reproduction (egg ratio), and survival of two common aquatic micro invertebrates: the rotifer species *Brachionus calyciflorus* and *Brachionus fernandoi*. The MPs were combined with food quantity, limiting and saturating food concentration, and with food of different quality. We found variable fitness responses with a significant effect of 3-µm PS on the population growth rate in both rotifer species with respect to food quantity. An interaction between the food quality and the MPs treatments was found in the reproduction of *B. calyciflorus*. PA and SiO_2_ beads had no effect on fitness response. This study provides further evidence of the indirect effect of MPs in planktonic rotifers and the importance of testing different environmental conditions that could influence the effect of MPs.

## 1. Introduction

Plastic pollution is continuously increasing and without effective control, it will become more and more serious in the future. Currently, about 60 to 80% of the litter material in the environment is plastic [1].Plastic litter has a broad size, ranging from large plastic fishing nets and fragments of containers to very small particles in the millimeter or micrometer range and down to nanoparticles below 1 µm. Microplastics (MPs) have been found virtually everywhere in both terrestrial and aquatic ecosystems such as rivers, lakes, and oceans [2,3]. Plastics can enter aquatic systems from waste water treatment plants [4], through surface runoff [5,6,7], or from being deposited through the air [8]. Many studies have reported that microplastics harm a wide variety of aquatic organisms: the ingestion of large amounts of microplastics by aquatic organisms can reduce energy reserves and can affect growth and reproduction, which consequently increases the mortality of, for example, crustaceans [9], fish, mollusca, anellida[10]. The uptake of MPs from even smaller zooplankton can make them more available to larger taxa [11]. However, evidence supporting a quantitative risk assessment for microplastics is still missing due to a lack of method standardization and result ambiguity [12].A study from Sun et al. [13] showed that small-sized microplastics (0.07 µm; 0.05 µm) decreased rotifer survival and reproduction, whereas large-sized microplastics (0.7 and 7 µm) had no effect on rotifer life history traits. In contrast, Xue et al., [14] showed that larger microplastics (10–22 μm), in association with the algal food of similar size, suppressed the reproduction of rotifer, and this negative effect could be alleviated by increasing the food supply. Similar discrepancies have been found in studies conducted with the microcrustacean *Daphnia* [15,16]. Such discrepancies can result from different experimental set-ups, different shapes and types of plastics, and their relationship with food availability or food-size selection. Because of the shapes, size, and polymer composition of microplastics, there is still a necessity to better understand the effect of microplastics on aquatic organisms. Representative forms of microplastics in the environment are fragments and fibers, while microspheres are found less often [17,18]. Fragments and fibers accounted for 60% of all types of MPs, even in remote areas such as Lake Hovsgol in Mongolia [19]. One relevant component of shape is “spikiness”. It was shown that spiky particles (e.g., filaments) and irregularly shaped particles (e.g., fragments) had showed a greater potential to harm animals than smooth particles such as spheres did, because spiky particles are more difficult to egest than smooth particles [20].

Rotifers are a widely distributed group of zooplankton that is present in all types of freshwater and brackish water bodies. They play an important role in aquatic food webs at the interface between primary producers and secondary consumers. As filter feeding organisms, rotifers have a very limited capability for food particle selection. Thus, rotifers cannot avoid the ingestion of plastic particles while they are feeding on natural food, such as algae. Therefore, rotifers are good model organisms for the study of and to understand how microplastic pollution influences aquatic ecosystems. Since field populations of rotifers are often resource limited [21,22,23,24], resource availability and natural fluctuation of algal growth should also be taken into account when estimating the risk of plastic pollution. We tested two closely related rotifers species, which were previously considered as one species, *Brachionus calyciflorus* and *Brachionus fernandoi*. These two species, even though they have a very similar morphology, exhibit different ecology and life history traits [25,26,27].

We used 1-, 3-, 6-, µm polystyrene beads (PS) because they are commonly used in toxicological studies of other organisms [28,29]. In addition, we used polyamide nylon fragments (PA) that were 5–25 µm in length because they are relevant in the field. As a non-plastic control, we used silica beads (SiO_2_) (3 µm), and as the positive control, we used a treatment without artificial particles (only food algae). The different artificial beads were offered together with food algae at limiting and saturating food concentrations [30]. Moreover, the effects of the different microplastics were tested in *B. calyciflorus* in association with a different algal diet of *Monoraphidium minutum* and *Cryptomonas* sp., which is considered to be a high-quality food that can be ingested by rotifers [31,32].

The aim of this study was to quantify and compare the effect of differently sized and shaped particles made of different materials. We hypothesized that (1) the ingested beads could induce a decrease in the growth rate and reproduction of brachionids, acting as non-nutritional particles and that (2) the effect of microplastics is influenced by the food quantity and food quality.

## 2. Materials and Methods

### 2.1. Cultivation of Organisms

We used two species of pelagic rotifers, *Brachionus calyciflorus* s.s. (strain USA) and *B. fernandoi* (strain A10; [26]). Rotifers were raised in six well microtiter plates with sterile and vitamin-supplemented Woods Hole Culture Medium (WC) with saturating densities of *Monoraphidium minutum* (SAG 243-1, Culture Collection of Algae, University of Göttingen, Germany; ESD = 3.5 µm) as food. The phytoplankton species *Cryptomonas* sp. (Culture collection Göttingen, strain SAG-26-80; ESD = 5.9 µm [33]) was used as additional food in the food quality experiments [26]. Cultures were kept at 20 °C in a light–dark cycle of 14:10 h and at a light intensity of 35 µM photon s^−1^ m^−2^ photosynthetic active radiation (300–700 nm). Prior to the experiment, the rotifers were sieved through a mesh (30 µm) and were rinsed with sterile culture medium in order to separate them from their food. The carbon content was determined by an elemental analyzer (Euro EA 3000, HEKAtech Gmbh, Wegberg, Germany).

### 2.2. Microplastics

We used polystyrene microspheres (PS) of three different diameters as the microplastic beads in this study: 1.03, 3.06, and 5.73 µm (Polysciences, Inc. Fluoresbrite^®^ YG Polystyrene Microspheres, Warrington, USA); for convenience, we refer to them as 1-, 3- and 6-PS. A stock solution was prepared with deionized MilliQ water under sterile conditions to minimize bacterial growth. To keep the beads as singular particles, each stock solution was sonicated for 30 min and was mixed using a vortexer. Stock suspensions of silica (SiO_2_) beads in the size of 3.0 (cat. #SiO_2_-F-3.0) were purchased from microParticles GmbH (Berlin, Germany). The stock solution was prepared using the same methods as the one prepared for the PS beads. Nylon fragments (5–25 μm) were prepared by size fractionating polyamide nylon-6 powder (nylon, PA) (Goodfellow; AM306010) with 25 μm cellulose filter (Whatman^®^ qualitative filter paper, Grade 4) and 5 μm nylon mesh under a laminar flow hood. Prior to use, the microplastics were exposed to UV-light for 20 min to avoid bacterial contamination. For quantification, the fragments were suspended in ultrapure water and were analyzed with an electronic particle counter (CASY Schärfe System GmbH, Reutlingen, Germany) to assess the concentration and the total volume; moreover, a subsample was inspected using microscope, and the stock concentration and size range was assessed (Figure S2). The PS microbeads, the silica beads, and the PA fragments used in the present study have been previously used in numerous studies determining the effect and the ingestion of microplastics in pelagic and benthic organisms [28,29,34,35].

### 2.3. Experimental Procedure

For the population growth experiments, the two rotifer species fed on two carbon concentrations (0.5 mg C L^−1^, “Limiting food concentration” LF and 2 mg C L^−1^ “Saturating food concentration” HF, Table S1) of *M. minutum* in combination with 1, 3, 6 PS beads, three types of SiO_2_ beads, and 2 mg/L PA fragments with four replicates (Table S2). In this study, we used the same total amount of plastic (or silica) material, i.e., smaller particles were provided in higher numbers than larger particles.

In the second experiment, only the rotifer species *B.calyciflorus* was fed with a mix of algae species: *M. minutum* and *Cryptomonas* sp. Two carbon concentrations (0.5 “LF” and 2 mg C L^−1^ “HF”) were used. Both algal species were supplied in 0.25 mg C L^−1^ for LF and 1 mg C L^−1^ for HF, respectively. *B. fernandoi* was not exposed to the mixture of algal food because it became mictic, i.e., it switched to sexual reproduction when fed with the mixed diet.

The experiment was conducted in 6-well microtiter plates at 20 °C in the dark to avoid additional algal growth. In the beginning, 10 individuals were randomly chosen from the stock culture and were pipetted into each well filled with 10 mL of the respective food suspension. At intervals of 24 h, the animals (live and dead) and their eggs were counted in each well. When the populations increased, 10 live individuals were randomly picked and transferred into new wells daily, receiving fresh food suspensions. In a case where less than 10 individuals survived, all of the remaining animals were transferred. The experiment lasted for 10 days (there was the exception of one replicate from *B. fernandoi* at low food concentration that got lost). Microtiter plates were placed on a rocker (Bio-Rad, Double Rocker, Labnet International Inc., Woodbridge, NJ, USA) to reduce the particle sedimentation. For each replicate the intrinsic growth rate (*r*), the egg ratio (*m*; eggs/female), and the survival (*l*) per day (*t*) were calculated on a daily basis using the following equations [36,37,38]:

(1) $$r=ln\left(N_{t}\right)-ln\left(N_{t-1}\right)\;$$

(2) $$m=\frac{H_{t}}{N_{t}}\;$$

(3) $$l=1-\frac{D_{t}}{N_{t-1}}$$

where *N*_(*t−*1)_ is the initial number of individuals and where *N_t_*, *H_t_*, and *D_t_* are the final numbers of individuals, total eggs, and dead, respectively, on consecutive experimental days. The population growth rate (d^−1^) of each replicate as well as reproduction (eggs ind^−1^ d^−1^) and the probability of survival (d^−1^) were calculated by averaging *r*, *m*, or *l* of consecutive experimental days.

### 2.4. Statistical Analysis

To compare the results from different experiments, we used the intensity of growth rate reduction (Δ*r*) relative to the control group. The intensity of the growth rate reduction (Δ*r*) was expressed as the difference in the per capita population growth rates with and without microbeads; a measure often used in food limitation experiments follows [21,23,24,39,40]:

(4) $$\Delta r=r_{c}-r_{s}$$

where *r_c_* is the per capita population growth rate in the experiment without microbeads (control), and *r_s_* is the growth rate with the microbeads. A statistically significant growth reduction was present if the 95% confidence limits did not include zero and if the confidence intervals did not overlap. The effect of plastics and the interaction of food quantity, food quality, and plastics on the egg ratio and percentage of survival was analyzed using three-way ANOVAs and a pairwise comparison (Emmeans test) grouped by food against the reference group “control” with Bonferroni adjustment. The egg ratio was square-root transformed, and the percentage of survival was Yeo–Johnson transformed (lambda = 4.99) with the R-package “bestNormalize”. Normality was assessed graphically using QQ-plot, and the homogeneity of variances was assessed using Levene’s test. All of the statistical analyses were performed, and graphs were generated using R software (version 1.1.383).

## 3. Results

### 3.1. Effect of the MP Beads on Population Growth Rate

*Brachionus calyciflorus* and *B. fernandoi* experienced significant population growth rate reductions when exposed to the PS beads (Figure S5). Otherwise, there were no significant growth rate reductions in the treatments using PA fragments and silica beads (Figures S1, S3, and S4 showing ingested polymers).

**Figure 1:**
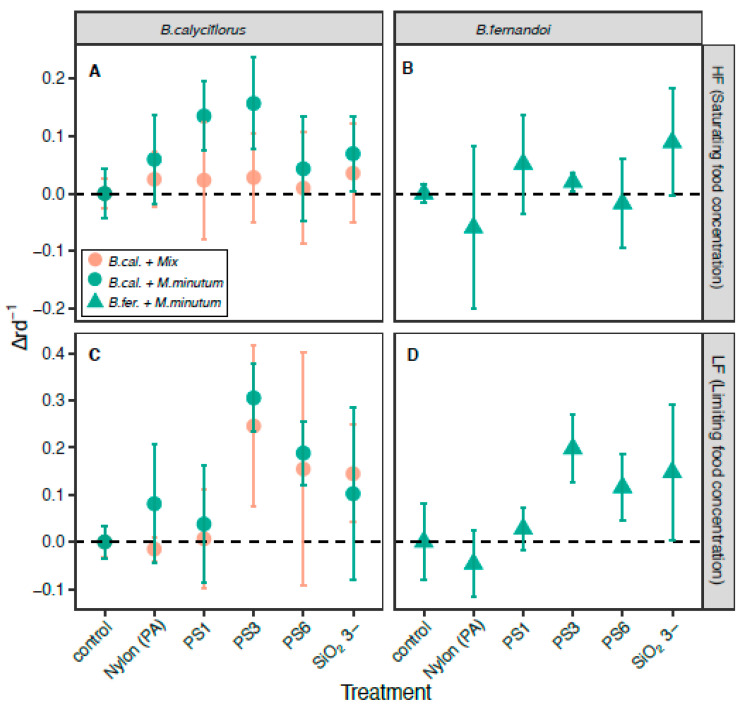
Intensity of food reduction (Δ*r* ± 95% confidence interval (CI)) of the rotifer *B. calyciflorus* and *B. fernandoi* at high and low food concentrations; (**A**–**C**) the red circles refer to the experiment with *B. calyciflorus* and the mixed algal diet (*M. minutum* and *Cryptomonas* sp.), and the green circles refers to the experiment with *B. calyciflorus* and one algal species (*M. minutum*); (**B**–**D**) the green triangle refers to *B. fernandoi*.

In detail, we found a significant growth rate reduction when *B. calyciflorus* was only fed on the *M. minutum* algae with the 1-µm PS beads (Δ*r* = 0.14; CI = 0.061) and 3- (Δ*r* = 0.16; CI = 0.079) at the saturating food concentration. For the limiting food concentration, we found significant growth reductions with the 3- (Δ*r* = 0.31; CI = 0.072) and 6-µm beads (Δ*r* = 0.19; CI = 0.067). Contrarily, when a mixed algal diet was provided to *B. calyciflorus*, no growth rate reduction was found at the saturating food concentration, and the rotifers showed a significant decrease in growth rate for the limiting food concentration for particles that were 3 µm in size (PS: Δ*r* = 0.25; CI = 0.171; silicate Δ*r* = 0.14; CI = 0.103). In a similar manner, *B. fernandoi* exhibited no growth rate reductions at the saturating food concentrations, and only exhibited reductions when exposed to the limiting food concentration and to the 3-µm PS beads (Δ*r* = 0.20; CI = 0.071), where we found a significant decrease in growth rate (Figure 1).

### 3.2. Effect of the MP Beads on Reproduction

**Figure 2:**
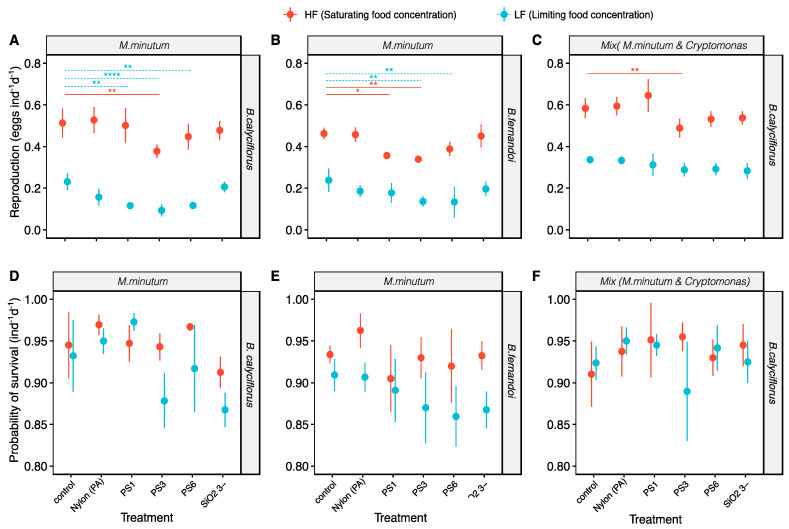
A−B−C egg ratio of *B. calyciflorus* and *B. fernandoi* exposed to the microbeads (mean ± SD); (**A**) egg ratio from *B. calyciflorus* fed on one algal species (*M. minutum*), with a statistically significant difference between the control group and the microbead treatment group; (**B**) egg ratio from *B. fernandoi* fed on one algal species (*M. minutum*), with a statistically significant difference between the control group and the microbead treatment group; (**C**) egg ratio from *B. calyciflorus* fed on mix algal diet (*M. minutum* and *Cryptomonas* sp.), with a statistically significant difference between the control group and the microbead treatment group; D−E−F percentage of survival of *B. calyciflorus* and *B. fernandoi* exposed to the microbeads (mean ± SD); (**D**) survival of *B. calyciflorus* fed on one algal species (*M. minutum*), with a statistically significant difference between the control group and the microbead treatment group; (**E**) survival from *B. fernandoi* feeding on one algal specie (*M. minutum*); (**F**) survival from *B. calyciflorus* fed on mix algal diet (*M. minutum* and *Cryptomonas* sp.), with a statistically significant difference between the control group and the microbead treatment group.

*Brachionus calyciflorus* and *B. fernandoi* responded similarly regarding the production of eggs per individual (F_1137_ = 1.3, *p* = 0.26; Table 1 and Figure 2).

The egg productions were affected by the food concentration (F_1137_ = 997.0, *p* < 0.0001; Table 1), the different algal diets (F_1137_ = 125.5, *p* < 0.0001; Table 1), and the plastic treatments (F_5137_ = 20.3, *p* < 0.0001; Table 1). Moreover, the effect of the food concentrations on the egg ratio differed between the two rotifer species (F_1137_ = 16.6, *p* < 0.0001; Table 1) and between the two algal diets within the same species (F_1137_ = 33.5, *p* < 0.0001; Table 1). Regarding the effect of the plastic treatments, in general, we did not find significant changes after limiting the saturating food concentration (F_5137_ = 1.0, *p* = 0.42; Table 1); on the contrary, the effect varied between the two algal diets (F_5137_ = 4.23, *p* < 0.01; Table 1). The rotifers responded differently depending on the plastic treatments, but no significantly different effect was found between the control group and the rotifers exposed to PA fragments and silica beads. A reduction in egg production was mostly found with the 3-µm PS beads, with the exception of the experiment with *B. calyciflorus* when limiting then food concentration in the mixed algal diet. *B. calyciflorus* was more vulnerable to a decrease in the egg ratio when fed on a monoculture diet and with PS beads when the food concentration was limited (LF: PS1, *p* < 0.01; PS3, *p* < 0.0001; PS6, *p* < 0.01; Table S3), and a minor vulnerability was also shown with the saturating food concentration (HF: PS3, *p* < 0.01; Table S3). When the mixed algal diet was provided, *B. calyciflorus* exhibited a less pronounced decrease in the egg ratio, with the only significant reduction only being seen with the 3-µm PS beads (HF: PS3, *p* < 0.01; Table S3). Similarly, *B. fernandoi* showed an eggs ratio reduction with PS beads at the saturating (HF: PS1, *p* < 0.05; PS3, *p* < 0.01; Table S3) and limiting food concentrations (LF: PS3, *p* < 0.01; PS6, *p* < 0.01; Table S3).

### 3.3. Effect of the MP Beads on Survival

The probability of survival was affected by the food quantity (F_1137_ = 28.6, *p* < 0.0001; Table 1) and plastic treatments (F_5137_ = 5.6, *p* < 0.001; Table 1) and differed between the two species (F_1137_ = 20.2, *p* < 0.0001; Table 1). The effect of the beads changed depending on the food concentration (F_5137_ = 3.9, *p* < 0.01; Table 1), on the algal diet (F_5137_ = 3.2, *p* < 0.01; Table 1), and on the species (F_5137_ = 3.3, *p* < 0.01; Table 1). Nevertheless, for the two species and the different algal diets, no significant differences were found between the control group and the beads.

## 4. Discussion

The aim of this research was to investigate and compare the effects of different sizes and types of microbeads and the role of food quantity and quality in a freshwater rotifer population. In this study, we highlighted the decrease of the population growth rate and reproduction (egg ratio) of two freshwater rotifer species, *Brachionus calyciflorus* and *Brachionus fernandoi*, in response to exposure to PS beads at the limiting food concentration. Moreover, *B. calyciflorus* exhibited reduced fitness when exposed to MPs with a single algal food species at the saturating food concentration. In contrast, the (PA) nylon fragments and the silicate beads had no effect on the population growth rate, egg ratio, and survival.

### 4.1. The Role of Food Quantity and Food Quality on Microplastics Effect

Our experiments showed that the population growth rates of the two rotifers species and with both algal diets were more affected at the limiting food concentration with the presence of the 3-µm PS beads. Only *B. calyciflorus* showed a reduction in the population growth rate at a high food concentration with the monoculture algal diet. In fact, the population growth rate of *B. calyciflorus* did not decline when a mixed algal diet was provided at the saturating food concentration; similarly, *B. fernandoi* only exhibited a reduced population growth rate at the limiting food concentration. In addition, the growth rate reduction was less pronounced in *B. calyciflorus* with the mixed algal diet than it was with the monoculture algal diet (Figure S1). The egg production was also mostly affected mostly by the PS beads; the effect of the microplastics, if present, was not influenced by the different food concentration but instead depended more on the algal diet provided to the rotifers. For instance, *B. calyciflorus* and *B. fernandoi* showed a reduced egg ratio at the limiting and saturating food concentrations, with different intensities, but when a mix algal diet was provided, *B. calyciflorus* only exhibited a reduced egg ratio with the 3-µm PS beads at the saturating food concentration and had no effect at the limiting food concentration. For *B. calyciflorus* at the limiting food concentration, we found an inverse relation between the population growth rate and the number of eggs produced, where the number of individuals decreased but not the number of eggs; in contrast, at the saturating food concentration, the number of eggs per individual declined, but not the number of individuals. Although the population growth rate and egg ratio are expected to be linked to each other, they do not match perfectly. On the one hand, at low food levels, animals can increase their life span at the expense of reproduction. In our experimental set up, this led to a lower growth rate reduction but to a strong decline in the egg ratio. On the other hand, at the maximal growth rates, a high number of not yet reproducing juveniles are part of the population, leading to sub-maximal egg ratios. Our findings are in accordance with Korez et al., [41] where a marine isopod was not affected by microplastics when they received a sufficient amount of food with a high nutritional quality. A surplus in the microplastics at a low food concentration caused a significant reduction in food uptake and digestive enzyme activities. One likely explanation for the decrease in egg ratio in rotifers that is connected to microbeads exposure, is the food dilution effects, which have been found in nematodes and crustacea [12,29]. Microbeads, which are mostly of the same size of the supplied food, interfere with normal food ingestion, and in addition, the particles act as a non-food item, providing no energy resource. Thus, the microbeads occupy space in the digestive tract, decreasing the available space for algal food. A similar study on cladocerans determined that chronic exposure to PS beads led to a reduction in the number of offspring, which could be explained by the downregulation of several digestive enzymes that can interfere with the animal´s nutrient supply and that can affect their fitness [42].

Food quality may be more important in the explanation of the variation in zooplankton fitness than food quantity [43]. The food quality acts on consumer physiology through morphological traits such as the shape as well as the nutritional value. This is evident for organisms such as rotifers, who strongly depend on dietary nutrient supply. A decrease in food supply may lead to a shift in energy allocation and less available energy, resulting in a decrease fitness response [44,45,46]. Our findings indicate no differences between the two species in terms of the egg ratio, but as in previous studies, the food quantity influenced the reproduction differently [38]. Previous studies demonstrated the importance of food quality effects on the population growth rate, fecundity, and survival [47] as well as the differences in the life history traits between *B. calyciflorus* and *B. fernandoi* feeding on different algal foods [38]. Divergence in other life history traits were found [27] between *B. fernandoi* and *B. calyciflorus* by Zhang et al. since *B. fernandoi* invests less in sexual reproduction and has a higher population growth rate than the others brachionids. In addition, *B. calyciflorus* has a higher heat tolerance than *B. fernandoi* [26].These findings support the finding that *B. fernandoi* and *B. calyciflorus* differ in their ecology and react to stressors in a different way.

### 4.2. Size Particles Effect

The population growth rate and reproduction of the two rotifer species was significantly reduced when exposed to 3-µm PS beads. The size of the 3-µm PS beads is close to the size of the food alga and is at the lower end of the efficiently used food-size spectrum in *Brachionus* species [48,49,50,51]. This can explain why an effect was only found for the 3- and 6-µm beads. Our results are in accordance with Xue et al., [14], who showed that the reproduction of rotifers was suppressed when they were exposed to polyethylene microbeads (10–20 µm) along with algal food of a similar size. In our experiment, the survival percentage was not affected by the presence of microbeads, even when exposed to 3-µm PS, which had the strongest negative fitness response.

Different results were found by testing very small, nano-sized PS particles (37 nm, 0.07 µm) in marine brachionids, where the population growth rate decreased by more than 50%. On the contrary, large-sized PS beads had no effect on the population growth rate and reproduction [13]. The different results could be related to the different feeding efficiencies of the rotifer species. Furthermore, the nano-sized plastic beads mostly interfered at the cellular level. Micro- to medium-sized particles, similar to those in the present study, and particles that are up to 20 µm in size might interfere with the feeding and may dilute the food; in addition, large particles seem to have no effect on micro-zooplankton because they are non-edible food for them [48,49,50,51].

### 4.3. Silica and (PA) Nylon Microbeads

No effect on the fitness response was found when the rotifers were exposed to silica beads and polyamide fragments. The concentration and the specific density of the material play an important role in the uptake of particles in rotifers and could be a likely explanation for our findings. In fact, silica beads and the polyamide (PA) have a higher specific weight and a higher sinking velocity than PS. To prevent sedimentation, we applied agitation, but the ingestion process itself might have been affected by the weight. One may speculate that heavy particles are difficult to ingest. In the natural environments, animals are exposed to particles along with other suspended solids. A number of studies found no negative effects on the fitness of rotifers when they were exposed to suspended clay, whereas cladocerans were affected by clay particles [52,53]. Although rotifers and cladocerans are typical filter feeders, rotifers can feed more selectively, and they were able to avoid ingesting clay particles [52,53]. These results suggest that rotifers might be less affected by plastic pollution than cladocerans. Studying the effect of irregularly shaped MPs, *D. magna* was more affected by MPs than by mineral particles of a similar size, potentially leading to extinction within one and four generations [44,54,55]. A mechanism counteracting the ingestion of fragments is aggregation, which leads to particle sizes that are unable to be digested [20,49]. Until now, no general conclusion can be drawn as to which factors drive the ingestion and impact the size, shape, weight, and type of plastics on animals: Klein et al. [56] have recently found that the ingestion of beads and fragments in freshwater shrimp was more influenced by the size of the particles than by their shape, whereas the ingestion was not influenced by the presence of the food. Copepods, instead, ingest more fragments than beads or fibers [57]. Marine off-shore zooplankton ingested more fragments than the ones close to the urban coast [58]. These findings suggest a strong particle type and a species-specific role.

### 4.4. Ecological Relevance

A crucial issue in the research on plastic pollution is that the detection of particles becomes more and more difficult with decreasing size. At the moment, there is no method available that can reliably quantify microplastics in the size range used in this study in natural water samples with algae, bacteria, and detritus. The concentration of the smallest MPs size (<10 µm) cannot be estimated at present, but from modelling studies, it is likely that the number of MPs in the environment increases when the size decreases [59]. For instance, the number of particles in marine environment and freshwater sediment has been underestimated due to technical limitation [60,61]. At the time of the study, the concentrations of microbeads were, most likely, higher than the ones in the field; however, with increasing production and fragmentation, the amount of small microplastics will increase continuously.

Typically, laboratory conditions are chosen to match the needs of the test species as well as possible. In contrast, in the field, environmental conditions are highly variable over time and are often suboptimal in terms of temperature or food supply. In particular, food supply can vary strongly from low to high and vice versa over the course of mere days [62]. Under such suboptimal conditions, when animals are already stressed, the effects of pollutants can be stronger than they would be under ideal conditions, as demonstrated in the present study. Furthermore, the PS beads used for the experiment do not contain plasticizer or additives since they are used for standard tests. In fact, the polymer type and the chemicals that they contain can contribute to the toxicity of microplastics, creating an additional stress [63]. Indeed, one single plastic product can contain hundreds of chemicals [64]. These include additives such as antioxidants, flame retardants, plasticizers, and colorants as well as residual monomers and oligomers and side products of polymerization and compounds and impurities [65]. Once taken up, these plastic chemicals can have negative impacts. For instance, aqueous leachates from epoxy resin or PVC plastic products can induce acute toxicity [66] and alter life history traits [67] in *Daphnia magna*. Still, studies on the contribution of plastic chemicals to microplastic toxicity are scarce. Studies testing for the combined effects of more than two factors are generally rare [68]. In a study with *Daphnia*, Hiltunen et al. [69] tested for temperature, food quality, and microplastics. Using lower plastic concentrations, as was also the case in our study, they found that decreased food quality had the biggest effect on life history, and the low plastic concentrations had no effect. In another study, increasing the food quantity disproportionately reduced the uptake of MP, and no effect on *Daphnia* life history was found [70]. However, some results only become apparent after long-term exposure [71]. Combining these results, food quantity and quality have a strong impact on consumer life history that can be enhanced by high microplastic pollution.

## 5. Conclusions

Our study reveals that the negative effect of microplastics on a common freshwater invertebrate depends on the environmental conditions, which in this study, were food quality and quantity. This is one reason for the differing results in microplastic research and requires more attention in terms of plastic risk assessment. In addition, although standardized toxicological tests provide useful information on the toxic potential of pollutants, more realistic studies with various environmental conditions are needed to obtain deeper and more comprehensive insights on the problem of plastic pollution.

## Figures and Tables

**Table 1:** Results of three-way ANOVAs using square-root transformed data on the egg ratio and Yeo–Johnson transformed data on survival (lambda = 4.99) for the two rotifer species (*Brachionus calyciflorus* and *Brachionus fernandoi*) and the two algal diets (*Monoraphidium minutum*; *Monoraphidium minutum* + *Cryptomonas* sp.). The two species were provided with two quantities (0.5 and 2.0 mg C L^−1^) of *Monoraphidium minutum*. *B. calyciflorus* was provided with the same food quantities of a mixture of *Monoraphidium minutum* and *Cryptomonas* sp. as food.

	Egg-Ratio			Probability of Survival		
*Independent variables*	Df	F-Value	*p*-Value	Df	F-Value	*p*-Value
Alg	1137	125.5	<0.0001	1137	0.4	0.534
food	1137	997.0	<0.0001	1137	28.6	<0.0001
food × Alg	1137	33.5	<0.0001	1137	2.8	0.099
food × Treatment	5137	1.0	0.422	5137	3.9	<0.01
Specie	1137	1.3	0.258	1137	20.2	<0.0001
Specie × food	1137	16.6	<0.0001	1137	2.4	0.126
Specie × food × Treatment	5137	1.5	0.190	5137	0.6	0.699
Specie × Treatment	5137	0.3	0.907	5137	3.3	<0.01
Treatment	5137	20.3	<0.0001	5137	5.6	<0.001
Treatment × Alg	5137	4.2	<0.01	5137	3.2	<0.01

## Data Availability

The data presented in this study are available upon request from the corresponding author.

## Supplementary Materials

The following are available online at https://www.mdpi.com/article/10.3390/toxics9110305/s1, Figure S1: Population growth rate, Figure S2: Size range distribution of PA nylon beads, Figures S3 and S4: PA beads ingested by *B. calyciflorus*, Figure S5A,B: PS beads ingested by *B. calyciflorus*. Table S1: Concentration of food algae, Table S2: Concentration of microbeads, Table S3: Results from the Emmeansߣ test.
