# Supplementary material for: Variable Fitness Response of Two Rotifer Species Exposed to Microplastics Particles: The Role of Food Quantity and Quality
Source: Toxics. 2021 Nov 13;9(11):305. doi: 10.3390/toxics9110305 (PMC8619062; doi:10.3390/toxics9110305)
Supplement: Supplementary file 1 [file toxics-09-00305-s001.zip › toxics-1451395-supplementary.pdf]

toxics

Supplementary Materials: Variable Fitness Response of Two Rotifer Species Exposed to Microplastics Particles: The Role of Food Quantity and Quality

Claudia Drago and Guntram Weithoff

Table S1. Concentration of food alga used for the experiments at saturating food concentration and limiting food concentration. The concentrations are expressed in cell/ml and correspond to 2 mgCL^{-1}.

**Saturating food concentration (HF) (cell/ml)**		
B. calyciflorus* & *B. fernandoi	***B. calyciflorus***	
M. minutum	*M.minutum*	*Cryptomonas*
2.07×10^5	1.04×10^5	9.98×10^3
**Limiting food concentration (LF) (cell/ml)**		
B. calyciflorus & *B. fernandoi*	*B. calyciflorus*	
M. minutum	*M.minutum*	*Cryptomonas*
5.18×10^4	2.59×10^4	2.50×10^3

Table S2. Concentration of the microbeads used in the experiments. The concentrations are expressed as number of plastics per ml and correspond to 2 mg/L.

**Plastic (p/ml)**				**Silicate**
**PS**			**PA**	**SiO$_2$**
1 µm	3 µm	6 µm	5 µm - 25 µm	3µm
3.33×10^6	1.27×10^5	1.93×10^4	1.00×10^3	7.96×10^4

Table S3. Results from the pairwise comparisons (Emmeans test) relative to the egg ratio (Repr.t) of *B.calyciflorus* + one algal species, *B. fernandoi* + one algal species and *B.calyciflorus* + mix algal diet.

Brachionus calyciflorus* + *M. minutum

food	term	.y.	group1	group2	df	statistic	*p*	*p*.adj	*p*.adj.signif
HF	Treatment	Repr.t	control	nylon (PA)	44	0.72739	4.71E-01	1	ns
HF	Treatment	Repr.t	control	PS1	44	1.26585	2.12E-01	1	ns
HF	Treatment	Repr.t	control	PS3	44	3.8928	3.32E-04	0.00166	**
HF	Treatment	Repr.t	control	PS6	44	2.36431	2.25E-02	0.11271	ns
HF	Treatment	Repr.t	control	SiO2 3	44	1.71702	9.30E-02	0.46504	ns
LF	Treatment	Repr.t	control	nylon (PA)	44	2.42786	1.93E-02	0.09673	ns
LF	Treatment	Repr.t	control	PS1	44	3.95203	2.77E-04	0.00139	**
LF	Treatment	Repr.t	control	PS3	44	5.02562	8.86E-06	4.4E-05	****
LF	Treatment	Repr.t	control	PS6	44	3.88952	3.36E-04	0.00168	**
LF	Treatment	Repr.t	control	SiO2 3	44	0.73044	4.69E-01	1	ns

Brachionus fernandoi* + *M. minutum

food	term	.y.	group1	group2	df	statistic	*p*	*p*.adj	*p*.adj.signif
HF	Treatment	Repr.t	control	nylon (PA)	44	0.97434	3.35E-01	1	ns
HF	Treatment	Repr.t	control	PS1	44	3.26718	2.11E-03	0.01055	*

HF	Treatment	Repr.t	control	PS3	44	3.70227	5.92E-04	0.00296	**
HF	Treatment	Repr.t	control	PS6	44	2.51116	1.58E-02	0.0789	ns
HF	Treatment	Repr.t	control	SiO2 3	44	1.13056	2.64E-01	1	ns
LF	Treatment	Repr.t	control	nylon (PA)	44	1.6058	1.15E-01	0.57736	ns
LF	Treatment	Repr.t	control	PS1	44	1.92509	6.07E-02	0.30349	ns
LF	Treatment	Repr.t	control	PS3	44	3.39709	1.45E-03	0.00727	**
LF	Treatment	Repr.t	control	PS6	44	3.7665	4.88E-04	0.00244	**
LF	Treatment	Repr.t	control	SiO2 3	44	1.25057	2.18E-01	1	ns

Brachionus calyciflorus + *Mix algal diet*									
food	term	.y.	group1	group2	df	statistic	*p*	*p*.adj	*p*.adj.signif
HF	Treatment	Repr.t	control	nylon (PA)	44	-0.3798	7.06E-01	1	ns
HF	Treatment	Repr.t	control	PS1	44	-2.0795	4.34E-02	0.21715	ns
HF	Treatment	Repr.t	control	PS3	44	3.44998	1.25E-03	0.00624	**
HF	Treatment	Repr.t	control	PS6	44	1.83156	7.38E-02	0.36896	ns
HF	Treatment	Repr.t	control	SiO2 3	44	1.61583	1.13E-01	0.56639	ns
LF	Treatment	Repr.t	control	nylon (PA)	44	0.14795	8.83E-01	1	ns
LF	Treatment	Repr.t	control	PS1	44	1.2119	2.32E-01	1	ns
LF	Treatment	Repr.t	control	PS3	44	2.33196	2.43E-02	0.12172	ns
LF	Treatment	Repr.t	control	PS6	44	2.14551	3.75E-02	0.18735	ns
LF	Treatment	Repr.t	control	SiO2 3	44	2.62153	1.20E-02	0.05988	ns

The egg ratio was square root transformed and grouped by food against the reference group control. p values were adjusted with Bonferroni and significance is expressed as $p < 0.05$.

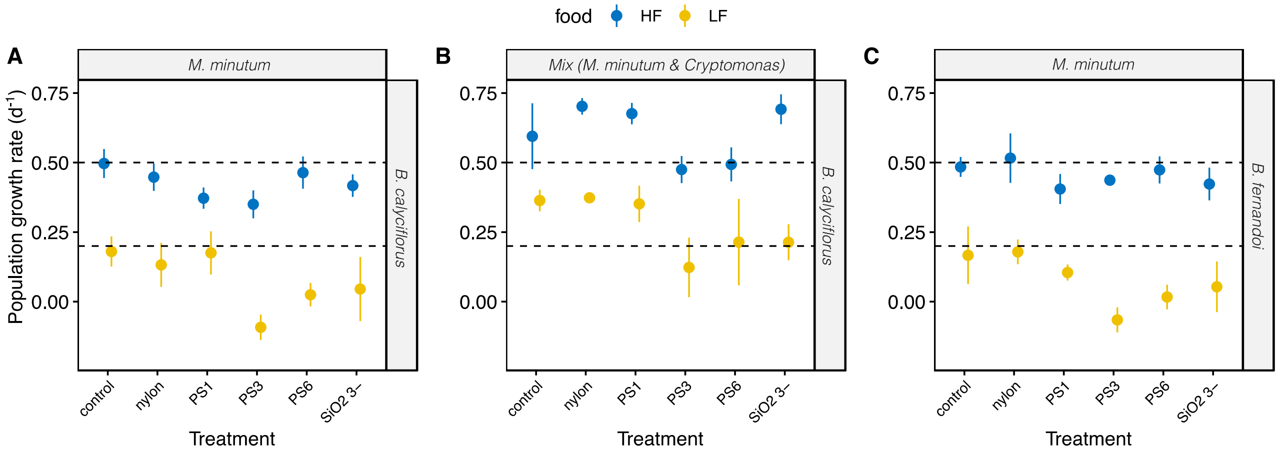

Figure S1. Population growth rate (mean ± SD) of *B. calyciflorus* (**a**) and *B. fernandoi* (**c**) with one algal species and mix algal diet for *B.calyciflorus* (**b**). Blue dots represent the population growth rate at saturating food concentration (HF) and the yellow dots are the population growth rate at limiting food concentration (LF).

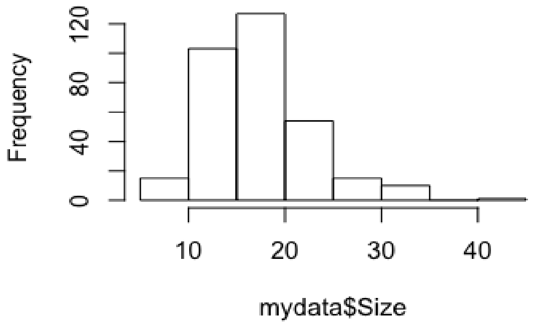

Figure S2. Size range distribution of the PA Nylon beads.

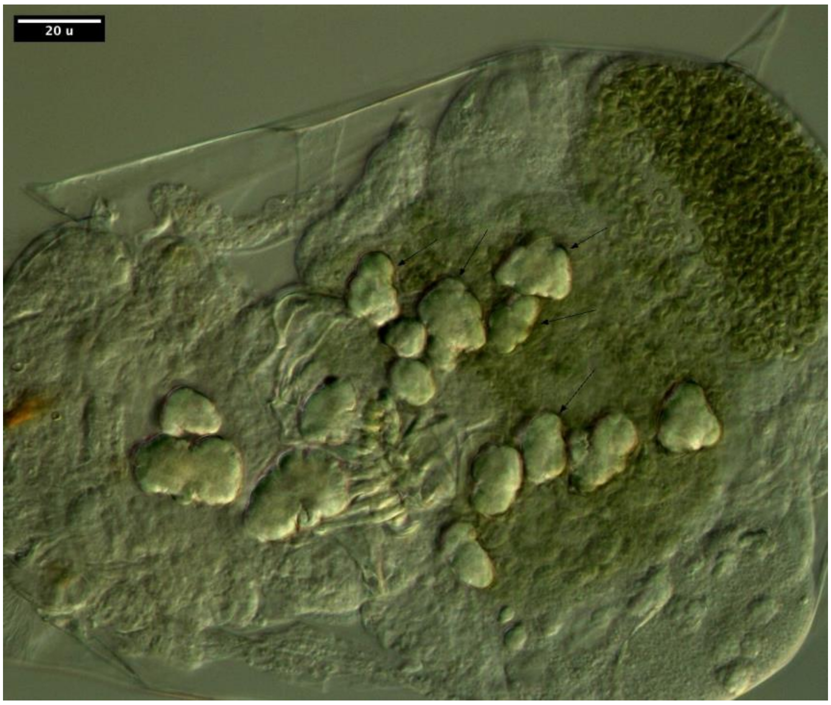

Figure S3. PA Nylon beads ingested by *B.calyciflorus*, the beads are indicated with the black arrows.

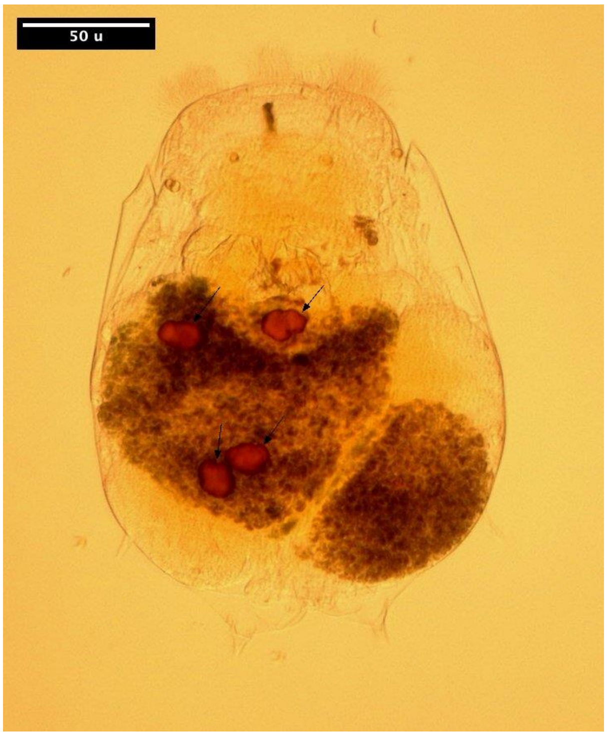

Figure S4. PA Nylon beads ingested by *B.calyciflorus* fixed with Lugol, the PA beads are indicated with the arrow.

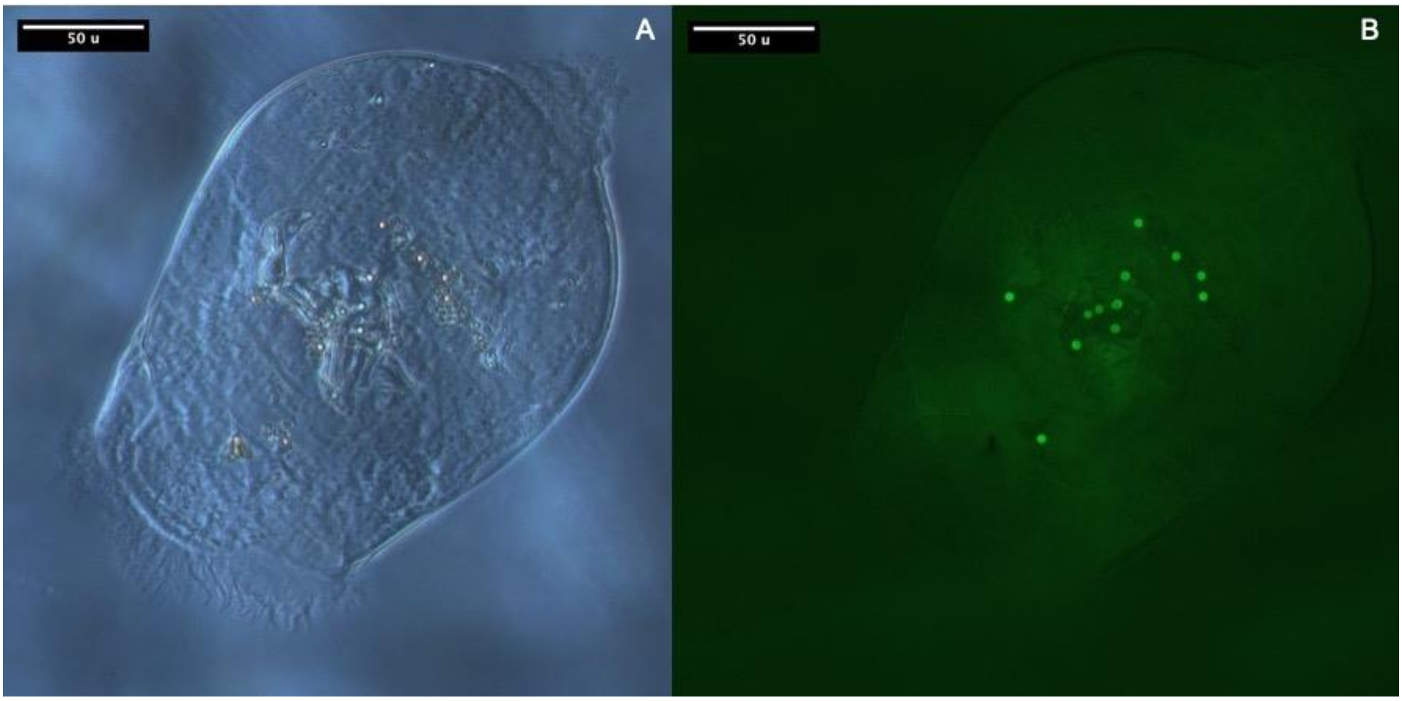

Figure S5A–B. PS beads (3µm) ingested by *B.calyciflorus* (**A**) in bright light and (**B**) fluorescent light.
